# Novel survey distribution methods: impact on antimicrobial resistance research outcomes

**DOI:** 10.1093/jacamr/dlae055

**Published:** 2024-04-23

**Authors:** Rasha Abdelsalam Elshenawy, Nkiruka Umaru, Zoe Aslanpour

**Affiliations:** Department of Pharmacy, School of Life and Medical Sciences, University of Hertfordshire, Hatfield AL10 9AB, UK; Department of Pharmacy, School of Life and Medical Sciences, University of Hertfordshire, Hatfield AL10 9AB, UK; Department of Pharmacy, School of Life and Medical Sciences, University of Hertfordshire, Hatfield AL10 9AB, UK

##  

### Introduction

Antimicrobial resistance (AMR) caused over 1.2 million deaths in 2019.^1^ AMR poses a significant global health crisis, with predictions of 10 million deaths per year by 2050.^2^ Recognized by the WHO as a top public health threat, AMR poses significant challenges, exacerbated by the COVID-19 pandemic.^3,4^ Antibiotic resistance research is paramount for understanding prescribing behaviours, AMR and antimicrobial stewardship (AMS), aiming to improve antibiotic prescribing practices and combat the threat of AMR effectively.^5^ Survey studies offer a valuable avenue for antimicrobial research, providing insights into prescribing behaviours, attitudes towards AMR, and the effectiveness of AMS practices.^6^ The field of antibiotic prescribing has encountered challenges in survey participation, which is evident at both local and national levels.^7^ Recruiting healthcare professionals to respond to surveys on antibiotic prescribing presents unique challenges due to their demanding schedules, the complex nature of antibiotic prescribing and the urgent need to address AMR.^8^ Therefore, innovative survey distribution methods are essential to enhance participation rates, gather comprehensive data and inform evidence-based interventions to mitigate the threat of AMR effectively.^9^

A research project was undertaken at one of the National Health Service (NHS) Foundation Trusts. A prospective survey questionnaire was conducted to investigate the perceptions, attitudes and knowledge of 240 healthcare professionals regarding antibiotic prescription practices during the pandemic. The survey was initiated on 12 June 2023, and concluded on 13 September 2023. The survey was administered using Qualtrics and distributed electronically, accompanied by an invitation letter, participant information sheet^10^ and professional poster. Ethical approval for this study was obtained from the Health Research Authority (HRA), with the Research Ethics Committee assigning reference number 22/EM/0161. In accordance with this approval, the study protocol underwent review and was approved by the University of Hertfordshire ethics committee under reference LMS/PGR/NHS/02975. Patient and public involvement were integral to this study, with representatives from the Citizens Senate being engaged. Their valuable input and feedback were integrated into the study's design and execution. Additionally, this study has been registered with the ISRCTN registry and Octopus, the global primary research record, ensuring transparency and accountability in research dissemination and reporting.^10,11^

The aim of this article is to evaluate the impact of using novel hybrid survey distribution methods, integrating traditional and digital strategies, in enhancing participation and achieving the target response for AMR/AMS research outcomes.

The objectives were as follows:

- To evaluate the effectiveness of novel survey dissemination strategies in healthcare settings;
- To explore the potential for applying innovative survey distribution strategies across various areas related to AMR/AMS research;
- To highlight the role of collaborative, multidisciplinary approaches in conducting impactful survey research to address public health challenges and enhance AMS efforts;
- To identify key insights and recommendations to inform future AMR/AMS research initiatives based on the findings.

### Methods

To secure the HRA's ethical approval,^12^ preparing attractive materials was crucial, including a visually attractive poster aimed at boosting respondent engagement. Specifically designed for healthcare professionals, this poster incorporated attractive colours, bold typography, QR codes and medical imagery. Such elements effectively captured the attention of doctors and nurses, encouraging their participation in a survey focusing on antibiotic prescribing and AMS practices during COVID-19. The poster's clear objectives and user-friendly layout further heightened its effectiveness as a tool for research participation (Figure 1).

**Figure 1.**
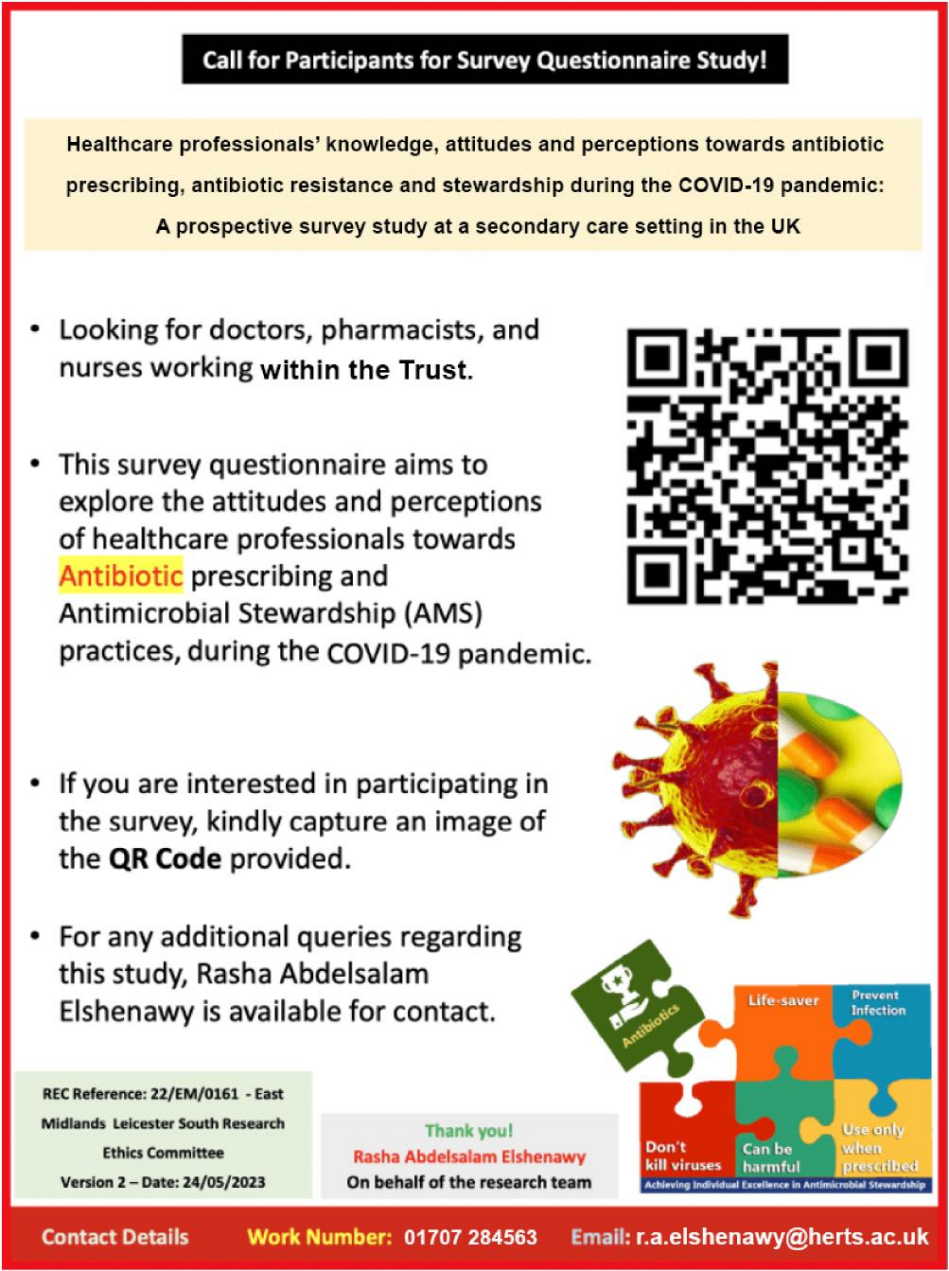
Professional poster for healthcare professionals within the Trust.

Innovative strategies significantly enhanced visibility and participation in a healthcare survey. QR code-enabled posters, designed with key survey information, were placed in high-visibility areas, such as nurses’ stations and staff rooms, ensuring easy access. The survey's digital distribution, through weekly e-newsletters and group e-mails from the Trust's Communication Team, was carefully timed to maximize engagement during optimal periods, such as early mornings, lunch hours and evenings. This multifaceted approach combined physical poster placement with effective digital dissemination, leveraging both traditional and modern methods. The R&D department's advice on poster sizes also played a crucial role, ensuring widespread and effective visibility across key hospital areas. This innovative mix of physical and digital strategies effectively increased survey participation among healthcare professionals in the wards, emphasizing the importance of varied and accessible distribution methods. To enhance survey participation, 50 A4-sized laminated posters were printed and strategically placed in hospital wards and departments. This was supported by digital dissemination through group e-mails and e-newsletters featuring QR codes, as shown in Figures S1–S3 (available as Supplementary data at *JAC-AMR* Online). The mix of traditional and digital methods significantly increased healthcare professionals’ involvement, boosting response rates and AMR awareness. Table S1 outlines a systematic distribution plan for a survey targeting healthcare professionals within a Trust. The approach includes targeted e-mails, strategic placement of posters, and digital distribution via newsletters and WhatsApp groups. This is designed to enhance participation rates in a study focused on antibiotic prescribing behaviours and AMS during the COVID-19 pandemic (see Supplementary data for details).

### Results

The high or improved participation in the survey was determined through a comprehensive analysis of various key indicators and outcomes resulting from the innovative survey dissemination methods implemented in this research project. Firstly, the response rate was monitored by comparing the number of responses received with the total number of surveys distributed over a specified period. Within a 3 month period, the objective of attaining the required sample size of 240 participants was successfully met, exceeding the response rate observed in preceding surveys or comparable questionnaires that did not incorporate innovative dissemination strategies. This achievement signifies an elevated level of participation, as highlighted by the R&D department. Secondly, qualitative feedback gathered from participants provided valuable insights into their experience with the survey dissemination methods. Positive feedback regarding ease of access, user-friendly design and the effectiveness of communication channels supported the notion of improved participation. Additionally, feedback from key stakeholders within the Trust, such as R&D directors and AMS leads, contributed to assessing engagement levels. Lastly, quantitative data on dissemination metrics, including the number of posters distributed (200 copies), frequency of digital distribution through group e-mails and electronic newsletters (e-newsletter), and the use of QR codes, offered tangible evidence of the reach and effectiveness of the distribution methods. These novel survey dissemination methods successfully sustained participation rates and improved the overall outcomes of antimicrobial survey research.

### Discussion

The research on AMR highlights the need for innovative survey distribution methods to address the AMR global health crisis effectively. Traditional and digital methods were combined to enhance participation rates among healthcare professionals. Visually appealing posters with QR codes were strategically placed in hospitals, complemented by digital dissemination through e-mails and e-newsletters. This multifaceted approach facilitated the achievement of the required responses within a 3 month period, in contrast to the response rates of previous surveys conducted within the same NHS Foundation Trust. Interestingly, the systematic review by Meyer *et al.*^13^ explores the variance in response rates for patient and healthcare professional surveys within surgery, revealing that factors such as survey type, follow-up methods, geographical location and respondent category significantly influence participation rates, with an average response of 70% for patient surveys and 53% for doctor surveys. The 2023 GP Patient Survey, conducted by NHS England and Ipsos, reveals insights into primary care service experiences, with a 28.6% response rate from 760 000 participants out of 2.65 million queried.^14^ However, there is a scarcity of data on survey participation rates in antibiotic prescribing, both locally and nationally.^13,14^ The innovative dissemination strategies presented in this article are pivotal, as they demonstrate a significant impact on AMR research outcomes by effectively engaging healthcare professionals—a key step in tackling AMR.

Effective survey distribution is crucial for reaching target audiences, securing higher response rates, and obtaining quality data. Pros include improved engagement through diverse methods, such as e-mail, social media and short message service texts, which can be tailored to specific audiences and geographical locations. Cons include the potential for low response rates if methods are not well chosen, data privacy concerns and the risk of surveys being perceived as spam. Balancing the selection of distribution methods against research project goals and audience preferences is essential for research success.^15^

In this article, the lead author (RAE) has developed a dissemination plan before initiating the survey distribution. This involved assessing various dissemination methods and considering their pros and cons in collaboration with the R&D team at the Foundation Trust. The most effective means of dissemination were chosen based on available resources and optimal communication strategies with the target healthcare professionals. Novel methods were employed, including the strategic placement of posters in hospital wards and on notice boards. QR codes were used to streamline dissemination, and there was a collaborative effort with the Trust’s Communication Team to distribute the survey through weekly e-newsletters and group e-mails.

Although traditional methods are capable of obtaining the necessary responses, they may face challenges in doing so and could result in significant delays in survey feedback, analysis and outcomes. Additionally, these methods sometimes struggle to engage a busy demographic effectively. Therefore, especially in the post-pandemic era, it is imperative to adopt innovative approaches, as detailed in the manuscript. These are essential for enhancing response rates, expediting survey responses and results, gathering complete data, promoting AMR research, and making a significant contribution to the global effort against AMR.^15,16^

As presented in this article, the use of traditional methods, such as sending reminder e-mails, was effective in encouraging responses and helped to increase survey participation. Qualitative feedback emphasized the effectiveness of these methods in facilitating participation. The combination of traditional and digital methods in disseminating the survey was also one of the important factors in achieving the required number of responses. This mixed approach offers benefits for resource-limited settings as it can be customized to fit each healthcare environment. Additionally, it encourages innovative thinking to reach the target survey participants and boost response rates effectively. Such engagement is invaluable in surveillance studies and other research related to AMR, a global health issue that requires urgent attention and continuous research to address and offer practical solutions to the AMR challenge.^17^ Furthermore, these innovative approaches to survey dissemination may be applied to a wide array of survey studies, extending well beyond the domain of antimicrobial resistance.^17^ They prompt the integration of creative, outside-the-box strategies that are tailored to engage the intended audience effectively. Such methods enable the adaptation of survey distribution to align seamlessly with the specific context of the healthcare environment, the distinctive characteristics of the survey itself and the available resources, thereby fostering a more impactful research engagement.^18^ Adapting these methods to fit the local context allows for the overcoming of barriers, ensuring effective communication and participation even where resources are scarce. This therefore extends the potential impact of AMR research to mitigate the AMR challenge and save patient lives.^18^

Following the survey analysis, the lead author also disseminated the final report to healthcare professionals at the Foundation Trust and shared research insights on Springer Nature Community,^19^ aligning with World Antibiotic Awareness Week.^20^ This effort highlighted the role of AMS against AMR, reflecting the United Nations’ Sustainable Development Goals.^19^

### Conclusion

This article has emphasized the impact of novel dissemination strategies in AMR/AMS research, demonstrating their effectiveness in enhancing participation and awareness among healthcare professionals. The strategic integration of hybrid methods—comprising traditional elements such as eye-catching posters with QR codes, and modern strategies like targeted e-mail campaigns—has effectively met participant targets, showcasing a viable solution to the challenge of engaging the busy medical community. This multifaceted approach not only shows the efficacy of these hybrid strategies in achieving the target survey responses but also highlights the potential of these methods to be applied broadly across various research domains beyond AMR. The findings advocate for a collaborative and multidisciplinary approach to conducting impactful antimicrobial research, emphasizing the urgent need for innovative, ‘outside-the-box’ methods in AMR/AMS research, particularly in the post-pandemic era. Although the surveys may not always be the most effective standalone research method, the innovative hybrid distribution strategies discussed could improve and complement other research methods. These strategies could significantly enhance AMR/AMS research, highlighting the need for creative, multidisciplinary approaches. This collaborative approach seeks to strengthen and sustain efforts that significantly support public health, ultimately aiming to save lives globally.

## Supplementary Material

dlae055_Supplementary_Data

## Data Availability

All data extraction forms, statistical analytic codes and any other materials used in the review are available on reasonable request.

## Supplementary data

Figures S1 to S3 and Table S1 are available as Supplementary data at *JAC-AMR* Online.
