# Supplementary material for: Novel survey distribution methods: impact on antimicrobial resistance research outcomes
Source: JAC Antimicrob Resist. 2024 Apr 23;6(2):dlae055. doi: 10.1093/jacamr/dlae055 (PMC11037271; doi:10.1093/jacamr/dlae055)
Supplement: dlae055_Supplementary_Data [file dlae055_supplementary_data.docx]

**Supplement Figures and Table**

**Figure S1.** A5 Professional Poster for Healthcare Staff: Displayed at Nurses' Stations, Staff Lounges, Medicine Trolleys, Main Workstations, MDT Rooms, Beside Doctors' Desks, and Notice Boards. Further placements include Medicine Rooms, IV Antibiotic Cabinets in Clean and Treatment Rooms, ensuring widespread visibility across an NHS Foundation Trust in the UK.


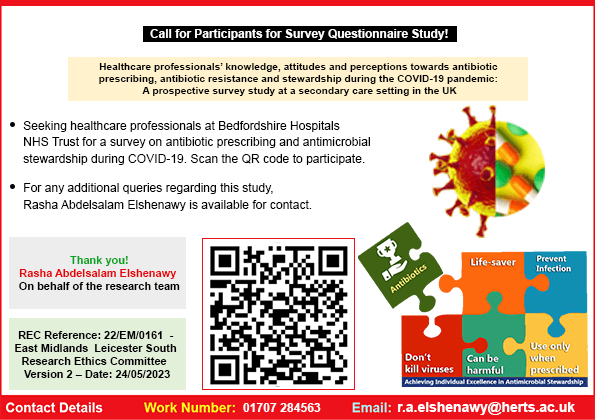


**Figure S2.** QR Code Distribution for Survey: Shared in Weekly Trust Newsletter and Group Emails to All Healthcare Professionals.

**
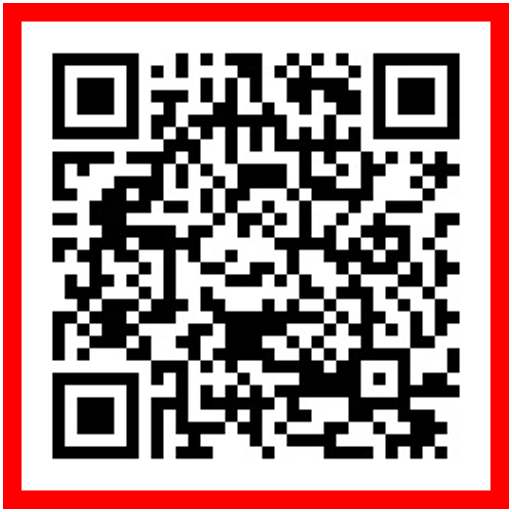
**

**Figure S3.** Maximising Survey Participation in AMR Research: Comprehensive Poster on Novel Distribution Strategies.


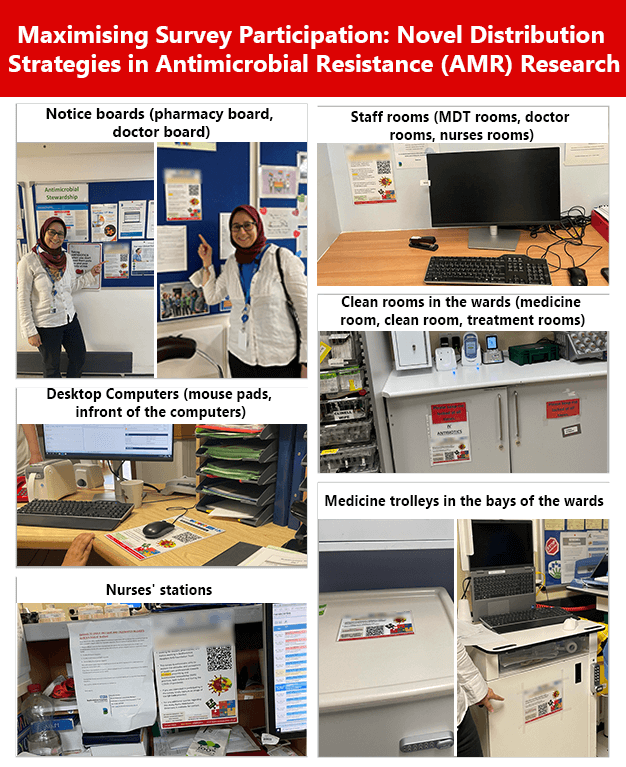


**Table S1.** Chronological order of the survey distribution among HCPs at the Trust.

| **Date** | **Survey Activity** |
| --- | --- |
| **12^th^ June 2023** | - A visit was made to the Trust to ensure the distribution of the invitation package by the R&D and AMS pharmacist, who sent an invitation email to the HCPs to invite them to participate in the survey initially. - The researcher had a meeting with the R&D department to discuss the survey distribution, poster design, and suitable printing sizes. - The R&D recommended printing two sizes: an A4-sized poster to be displayed in the wards and an A5 poster to be displayed on medicine trolleys. - Additionally, they recommended using laminated posters to avoid contamination within the wards. - Finally, they suggested designing another size for the poster to be used as a header in the invitation emails. They also recommended that the researcher distribute the survey and discuss the purpose of the survey with the staff in the wards to encourage responses. |
| **19^th^ June 2023** | - To promote survey responses, the researcher printed 50 A4-sized survey posters and requested that ward managers and the head of the department distribute them in the Trust. - The researcher attended the AMS round, discussing ways to distribute the survey within the Trust successfully with the AMS lead, microbiology consultant, and AMS pharmacists. - Doctors, nurses, and pharmacists were approached by researchers, who explained the survey's purpose and target population as a strategy to increase recruitment and distribution. A week later, an increase in the responses was recorded. - Survey posters were placed and displayed in several strategic locations in the Trust, such as nurses' stations, staff rooms, main halls, notice boards, and medicine trolleys in the bays. - One ward manager proposed placing the survey poster on the computer desk in the nurses' station. - Another sister in charge shared the poster within the nurses' WhatsApp group. A different ward manager forwarded the survey link to doctors and nurses using group emails. - The pharmacy cooperated by displaying the survey on the Antimicrobial Stewardship Clinical Board, the main notice board, and the digital pharmacy screen and by sending it to all pharmacists via email. |
| **22^nd^ June 2023** | - The AMS pharmacist at the Trust sent the in-survey invitation package to the pharmacists, while the R&D sent the invitation package to the doctors and nurses using group emails. - The researchers printed 50 copies of A4 and A5 posters and requested that the ward managers and head of department distribute them in the hospital. - The survey posters were placed and displayed in various strategic locations, such as MDT rooms, staff rooms, treatment rooms, doctor rooms, main halls, notice boards, nurses' stations, medicine trolleys in ward bays, and clean rooms at the Hospital. - One ward manager suggested placing the survey poster as a mouse pad for the main desktop computers in the wards. - Another sister in charge placed the poster in front of the desktop computer and on the counter. |
| **7^th^ July 2023** | - The communication team within the Trust sent the survey invitation package in the weekly newsletter, 'The Week,' to all HCPs within both hospitals. |
| **21^st^ July 2023** | - After two weeks, the communication team within the Trust re-sent the survey invitation package in the weekly newsletter 'The Week' to all HCPs within both hospitals. |
| **29^th^ July 2023** | - The R&D department resent the survey invitation package to the doctors, pharmacists, and nurses using the group emails. |
| **16^th^ August 2023** | - There was no increase in responses, possibly due to the summer holidays. - The researcher met with the supervisors to discuss methods to encourage survey responses. - They recommended waiting until September, when people return from summer holidays, and then resending the survey link. |
| **6^th^ September 2023** | - The researcher sent an email to the R&D and AMS pharmacists at both hospitals, as well as the AMS lead, requesting the re-circulation of the survey packages and suggesting the addition of a sense of urgency to the invitation email. - The researcher drafted a message with an urgent tone to be used for survey dissemination. - The researcher sent an email to the Communication Team within the Trust, requesting the inclusion of the survey links in the weekly newsletter 'The Week' and proposed using an attractive title: 'A Vital Call to Action'. |
| **13^th^ September 2023** | - As the number of responses decreased, the researcher communicated with the AMS pharmacist and the AMS lead in the Trust, suggesting they circulate the survey via the Trust's WhatsApp groups for pharmacists, doctors, and nurses. |
| **15^th^ September 2023** | - The data collection period has ended. |
